# Genetic characterization of Carbapenem-Resistant *Escherichia coli *from China, 2015–2017

**DOI:** 10.1186/s12866-021-02307-x

**Published:** 2021-09-17

**Authors:** Fengtian Li, Kun Ye, Xin Li, Liyan Ye, Ling Guo, Lifeng Wang, Jiyong Yang

**Affiliations:** Laboratory Medicine Department, First Medical Center of Chinese PLA General Hospital, Beijing, 100853 China

**Keywords:** *Escherichia coli*, Carbapenemase, NDM-5

## Abstract

**Background:**

The molecular characteristics of carbapenem-resistant *Escherichia coli* (CREco) remain unclear.

**Methods:**

We conducted a multi-center bacterial resistance monitoring project from 2015 to 2017.The minimum inhibitory concentrations ofCREco were determined bybroth microdilution method. The genome sequencing of CREcoisolates was performed, and single-nucleotide polymorphism (SNP) was analyzed.

**Results:**

A total of 144CREcoisolatescollected from 10 cities in China were involved in this study. ST167 (*n* = 43) is the most popular type, followed by ST410(*n* = 14), ST131(*n* = 9). There were 102 (70.83%) CREco isolates that produced various NDMs, including NDM-1 (*n* = 16), NDM-4(*n* = 1), NDM-5(*n* = 79), NDM-6(*n* = 2) and NDM-9(*n* = 4). In addition, 15 isolates produced KPC-2, three isolates wereIMP-4 positive, and three isolates produced OXA-48. Genetic relatedness and phylogenetic analysis showed that isolates with the same ST had a high degree of homology. Some STs (including ST167, ST410, ST131, ST46, ST405 and ST617) exhibited a trend of outbreak.

**Conclusions:**

The majority of CREco belonged to ST167, followed by ST410 and ST131, and most of them carried various NDM-coding genes. The spread of high-risk clones of CREco has occurred in different regions of China.

## Introduction

With the widespread of Extended-Spectrum β-Lactamases (ESBLs) in *Enterobacteriaceae*, the clinical efficacy of third-generations of cephalosporins,fluoroquinolones and aminoglycosidesin the treatment of ESBL-positive *Enterobacter* infection gradually decreased, which makes carbapenems have become thelasteffective antimicrobialagentsto control of infections caused by multi-drug resistant *Enterobacteriaceae* [1–3]. However, the emergence and spread of carbapenem-resistant *Enterobacteriaceae* (CRE) posed a serious threat to the health and medical safety of patients. Studies have shown that carbapenemases such as KPC, IMP, VIM and OXA-48 are the main mechanism of bacterial resistance to carbapenems [4].

Carbapenem-resistant *Escherichia coli* (CREco) is currently one of the main pathogens of CRE causing various clinical infections [5, 6]. According to a statistical result of CDC in the United States, the proportion of CREco was only 0.9% from 2006 to 2007, but it increased to 1.9% from 2009 to 2010 [1, 7]. In Europe, a recent survey showed that 19% of *E. coli* strains were CREco during 2013 to 2014 [2, 8]. In China, the monitoring results from 2004 to 2015 showed that the proportion of CREco remained at 0.8 to 3% during the ten years [3, 9].

NDM-1 was first discovered in 2009in a *Klebsiella pneumoniae* isolated from a Swedish patientwho had a hospitalization history in New Delhi, India [10], since then enterobacterial strains that produce NDM-1 spread widely in Asia and Europe, making NDM become one of the most prevalent resistance mechanism [4, 11–13]. Over the past decade, CREco have been increasingly reported worldwide. In addition, strains from different countries and regions also showed great differences in drug resistance mechanisms. For example, strains from Greece and Israel mainly produce KPC [8]. In Bulgaria and Denmark, NDM-producing *E.coli* (NDMEco) strains arethe most common, and OXA-48 is prevalent in Turkey [2, 8]. In China, the majority of CREco produces NDM [3, 9].

To date, there have been multiple outbreaks caused by various sequence type (ST) ofNDMEco. Among them, ST101, ST405, ST410, ST648, ST156, ST167 and ST131 are the most common clones worldwide [14]. In Europe, there are about 20 STs and ST101 is the most abundant clone, followed by ST410.InUnited States, the ST167, ST131, ST101, ST405 and ST617 were reported. About 38STs of NDMEcohave been identified in Asia, while ST101 was the most prevalent clone and has been found in Nepal, Pakistan and Korea. In addition, ST131 waspredominantly found in India, while ST167 was dominant in China [14]. Therefore, the most prevalent STs are important for identifyingoutbreak strains and controlling infections in different areas.

With the increase of carbapenem-resistant *E. coli* strains worldwide, long-term epidemiological surveillance and mechanisms research on the carbapenem-resistant *E. coli* are necessary for the global control of CRE. Recently, a three-year (2015–2017) monitoring of CRE was conducted at 10 hospitals across China, and 144clinical CREco isolates were collected. In this study, the epidemiological and genetic characteristics of these isolates were analyzed.

## Materials and methods

### Bacterial strains

Clinical enterobacterial isolates that showedresistance to any of the carbapenems were collected from 10representative hospitals across China during the monitoring period of 2015 to 2017including Beijing, Zhengzhou,Xian,Jinan, Shanghai, Shenyang, Guiyang, Chengdu, Guangzhouand Lanzhou, and were identified by VITEK MS (bioMérieux SA, Marcy-l’Etoile, France). 144 *E. coli* strains with decreased sensitivity to imipenem, meropenem, or ertapenem were screened. The specimens covered urine, sputum, blood, bile,drainage fluid, secretion, ascites, puncturefluid, pus, hydrothorax, and others. Since the clinical samples were collected during routine bacteriologic analyses in public hospitals, the ethical approval for the use of the clinical samples was not obtained. All data were anonymously analyzed.

### Antimicrobial susceptibility testing

The minimum inhibitory concentrations (MICs) of piperacillin-tazobactam,cefotaxime, ceftazidime,imipenem, ertapenem, meropenem, amikacin, sulfamethoxazole, tigecycline and polymyxinwere determined bybroth microdilution method using Biofosun®Gram-negative panels (Biofosun Biotech, Co., Ltd.,Shanghai, China). Results were interpreted according tothe interpretive standards of the Clinical LaboratoryStandards Institute guidelines (CLSIM100-S30), except for tigecycline, for which the European Committee on Antimicrobial Susceptibility Testing breakpoints (EUCAST, Version 11.0) was used to interpret the MICs. *E. coli* ATCC 25922 was used as the quality control strains.

### Whole-genome sequencing analysis

Genome DNA was extracted using the DNeasy® UltraClean® Microbial Kit (QIAGEN GmbH, 40,724 Hilden, Germany). The harvested DNA was detected by the agarose gel electrophoresis and quantified by Qubit 2.0 Fluorometer (Thermo Scientific). A total amount of 1 μg DNA per sample was used as input material for the DNA sample preparations. Sequencing libraries were generated using NEBNextUltraDNA Library Prep Kit for Illumina (NEB, USA) following manufacturer’s recommendations and index codes were added to attribute sequences to each sample. The whole genome was sequenced using Illumina NovaSeq PE150 at the Beijing Novogene Bioinformatics Technology Co., Ltd. Quality assessment were performed with Fastqc (Version 0.11.8), all reads-score above Q30 was used with follow-up analysis. After removingadapter, bar-code and trimming of the raw reads, sequences were assembled using SOAP denovo (SOAP Version 2.21) with default settings. N50, N90, coverage rate and scaffold number were used to identity denovo characters. Resistance genes, the multilocus sequence type (MLST) and virulence geneswere detected using Bacterial Analysis Pipeline (BAP) onCenter for Genomic Epidemiology website (http://www.genomicepidemiology.org/).

### Phylogenetic analysis

CSI Phylogeny 1.4 on Center for Genomic Epidemiology website was used in SNP finding and phylogenetic tree structuring. Whole genome SNPs derived from the core alignment were carriedout and processed in FigTree.v1.4.4 to generate amaximum-likelihood phylogenetic tree. The tree file was visualized by iTOLV.5 (https://itol.embl.de), and annotate information were edited byiTOL editor v1_1.

## Results

### Distribution of clinical CREco isolates

From 2015 to 2017, 144clinical CREco isolates were collected from 10 cities across China, including Beijing (*n* = 77), Zhengzhou (*n* = 14), Xian (*n* = 12), Jinan (*n* = 9), Shanghai (*n* = 8), Shenyang (*n* = 8), Guiyang (*n* = 4), Chengdu (*n* = 6), Guangzhou (*n* = 3) and Lanzhou (*n* = 3). The most common specimens were urine (*n* = 49, 34.03%), followed by sputum (*n* = 33, 22.92%), blood (*n* = 18, 12.5%), bile (*n* = 13, 9.03%), drainage fluid (*n* = 12, 8.33%), secretion (*n* = 5, 3.47%), ascites (*n* = 3, 2.08%), puncturefluid (*n* = 2,1.39%), pus (*n* = 2, 1.39%), hydrothorax (*n* = 1, 0.69%), and other (*n* = 6, 4.17%).

### Characteristics of clinical CREco isolates

Table 1 showed the susceptibilities of CREco isolates to antibiotics analyzed in this study. All isolates were sensitive to tigecycline, but 7 polymyxin-resistant isolates havebeen identified. The resistance rate ofcarbapenemase-producingCREco to imipenem and meropenem was higher than that ofcarbapenemase-negative isolates (*P* < 0.0001) according to chi-square test, and GraphPad.Prism.V7.0 was used for statistical analysis. The ST167 (*n* = 43) was the most popular type, followed by ST410 (*n* = 14), ST131 (*n* = 9), ST405 (*n* = 8), ST46 (*n* = 8), ST617 (*n* = 7), ST448 (*n* = 5), ST38 (*n* = 5), ST224 (*n* = 5), ST10 (*n* = 3) and ST354 (*n* = 3). In addition, each of the following STs contained two isolates respectively: ST12, ST361, ST457, ST648 and ST1193, while the remaining 20isolates belonged to 20 independent STs. There were 102 (70.83%) CREco isolates that produced various NDMs, including NDM-1 (*n* = 16), NDM-4 (*n* = 1), NDM-5(*n* = 79), NDM-6 (*n* = 2) and NDM-9 (*n* = 4). In addition, among the 144 isolates, 15 isolates produced KPC-2(10.42%, 15/144), 3isolates wereIMP-4 positive (2.08%, 3/144), and 3isolates produced OXA-48(2.08%, 3/144). The remaining 21isolates did not carry any known carbapenemase-coding genes which may due to production of the plasmid-encoded ESBLs in combination with outer membrane permeability defects [15]. Obviously, ST167 almost carrying the *bla*_NDM-5_ gene was the most common clinical strain type and it was widely distributed throughout the country in this study. ST410, which carries*bla*_NDM-5_ gene, seemed to be of greater concern since it was widely disseminated in China and known to cause infections nationwide. In addition, there were 7 NDM-producing isolates that carried *mcr-1* simultaneously.

**Table 1:** Percentage of CREco strains susceptible to antibiotics

Antibiotics	All strains (***n*** = 144)	Carbapenemase-producing strains (***n*** = 123)	Carbapenemase-negative strains (***n*** = 21)	***P*** value
AK	88.89	75.69	90.48	0.9735
PZT	0	0	0	0.0878
TGC	100	100	100	/
PB	95.13	95.93	90.48	0.2824
CAZ	0	0	0	0.6784
SXT	26.39	26.83	23.81	0.7717
CTX	0.69	0	4.76	0.2167
CIP	5.56	5.69	4.76	0.8636
ETP	0	0	0	/
IPM	13.89	4.88	66.67	< 0.0001
MEM	6.94	2.44	33.3	< 0.0001

*AK* amikacin, *PZT* piperacillin/tazobactam, *TGC* tigecycline, *PB* polymyxin, *CAZ* ceftazidime,

*SXT* sulfamethoxazole, *CTX* cefotaxime, *CIP* ciprofloxacin, *ETP* ertapenem, *IPM* imipenem,

*MEM* meropenem

### Genetic relatedness, **Virulence**Genes **and**SNP **phylogeny**

**Fig. 1:**
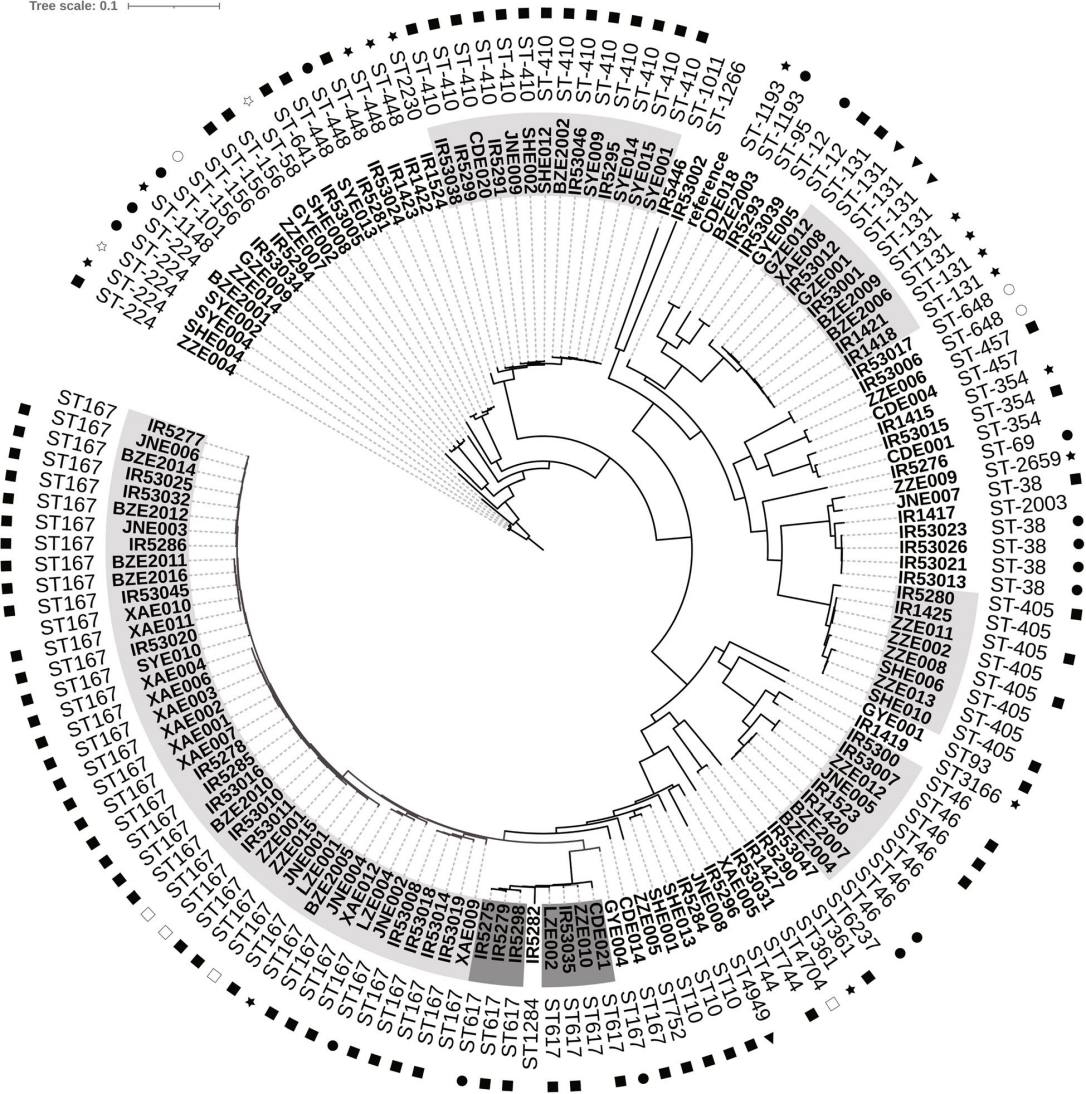
The SNP phylogenies of carbapenem-resistant*E.coli* isolates.:NDM-1;: NDM-4;:NDM-5;: NDM-6;:NDM-9;: KPC-2;:IMP-4;: OXA-48

**Fig. 2:**
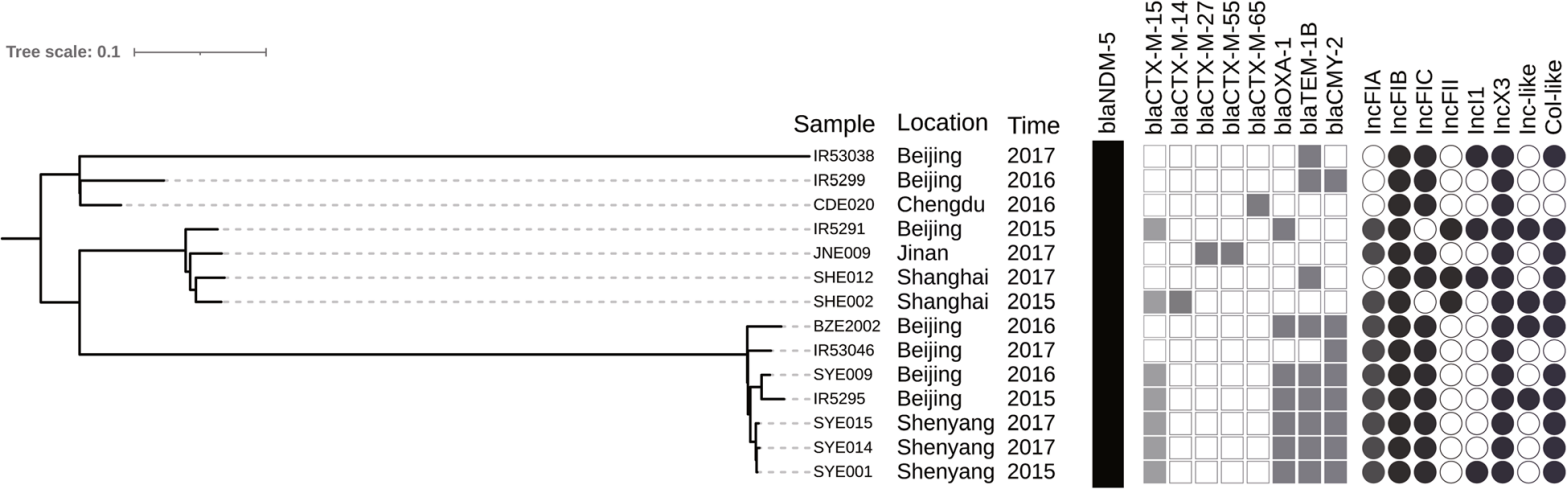
Phylogenetic distribution of ST410 *E.coli* isolates

**Fig. 3:**
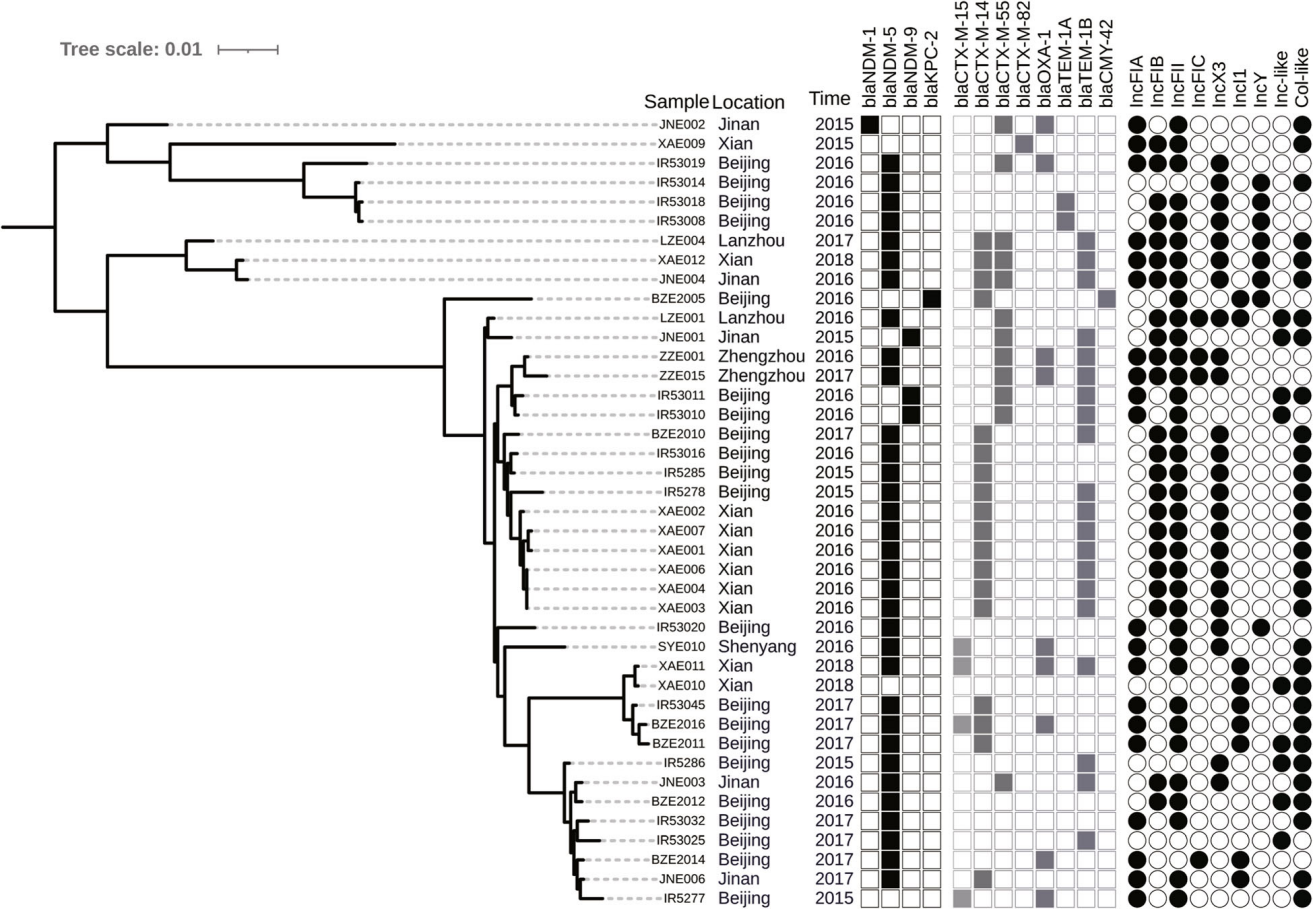
Phylogenetic distribution of ST167 *E.coli* isolates

Genetic relatedness and phylogenetic analysis showed that isolates with the same ST had a high degree of homology, and some STs (including ST167, ST410, ST131, ST46, ST405 and ST617) exhibited a trend of outbreak (Fig. 1). At the same time, the main virulence genesof main STs were identified, such asST167 (*capU*,*iss*),ST410(*gad*,*lpfA*),ST131(*iha,iss,sat*), ST46(*cma,gad*),ST405*(air,eil*A),ST617(*iss*). Moreover, isolates belonging to ST410and ST167were clustered into two distinct clades and sub-clades. Strains of each clades or subclades have been collected from different cities across China (Fig. 2 and Fig. 3).

Figure 2 showedST410 isolates carried *bla*_NDM-5_genefrom Beijing, Chengdu, Jinan, Shanghai and Shenyang, with 100% coverage of IncFIB and IncX3plasmids. Especially within subclade B2,the same resistance genes (*bla*_CMY-2_, *bla*_CTX-M-55_, *bla*_NDM-5_, *bla*_OXA-1_, *bla*_TEM-1B_) and plasmids (IncFIA, IncFIB, IncFIC, IncX3, Col) were detected in the isolatesSYE001, SYE009, SYE014 and SYE015.Meanwhile, BZE2002, IR5295 and IR53046 showed great similarity to the 4 isolates above expect forsmall difference ofbeta-lactamases and plasmids, so the 7 ST410 *E. coli* high risk clones spread in Shenyang and Beijing over 3 years.

The carbapenemase genes harbored in ST167 isolates covered Beijing, Xian, Zhengzhou, Jinan, Lanzhou and Shenyangpresented diversity (Fig. 3). Obviously, in subclade D2,the isolates BZE2010,IR5285,IR53016, IR5278, XAE001, XAE002, XAE003, XAE004, XAE006 and XAE007withthesame genes (*bla*_CTX-M-14_, *bla*_NDM-5_) and plasmids (IncFIB,IncFII,IncX3,Col) disseminated in Xian and Beijing. Furthermore, an outbreak of NDM-5-positive ST167 appeared in Xian.LZE004, JNE004 and XAE012with thesame genes (*bla*_NDM-5_, *bla*_CTX-M-14_,*bla*_CTX-M-55_,*bla*_TEM-1B_) and plasmids (IncFIA,IncFIB,IncFII,IncY,IncX3) spread among Lanzhou, Jinan and Xian.

## Discussion

In this study, CREco were mainly isolated from urine samples, which was consistent with a previous report about NDM-producing *E.coli*around the world [14]. In addition, our data showed that blood, bile and drainage of fluid also accounted for a considerable proportion of all samples, indicating that CREco was widely distributed in clinical practice and may cause multi-site infection, which should be strengthened management.

NDM-producing *E. coli* has been identified nationwide [16, 17], and production of NDM is the major mechanism of CREcofrom nationwide surveillance of clinical carbapenem-resistant*Enterobacteriaceae* strains in China during 2014 to 2015 [3, 9]. Data form Asia also showed that the majority of NDM-5-producing strains were identified in China [14]. In this study, 70.83% of CREco isolates produced NDMs, whileNDM-5-producing strains were dominant with an outbreakin 10 regionsacross the country. Selective pressure caused byincreased use of antibiotics may drive the evolution of NDM, leading to the emergence of its variants. NDM-5 has a stronger hydrolysis activity to carbapenems, meanwhile, NDM-5-producing strains can combine with other resistant mechanisms to mediate increased resistance to cephalosporins, quinolones and aminoglycosides [18],playing an important role in the emergence and spread of multi-drugresistant*E.coli* isolates.

It has been reported that most CREco belonged to ST101, ST405, ST410, ST648, ST156, ST744 and ST131 [19]. A recent multicenter study showed that the majority of CREco from China were ST131, and it was predicted that ST167 and ST410 seemed to be of greater concernsince they were widely disseminated nationwide [3, 9]. Our data confirmed this prediction. In this study, 29.86% (43/144) of CREcoisolates belonged to ST167, followed by 9.72% (14/144) of ST410 and 6.25% of (9/144) ST131, respectively. In addition, homology analysis showed thatthese high-risk clones have spread across different regions in China. Figure 3 showed that isolates of clade D2 carried *bla*_NDM-5_and spreadin 7 cities (Xian, Beijing, Jinan, Zhengzhou, Lanzhou, Shenyang, and Chengdu), indicating that ST167 played an important role in the transmission of the *bla*_NDM-5_ gene in China. It is particularly noteworthy thatCREco ST410 was a clone that was widely spread in some European countries,including Poland, Norway, Switzerland and the UK [14].Inter-regional mobility is likely to be an important route for the transmission of drug-resistant bacteria. In addition, ST410persists and/or causes recurrent infections in humans, including bloodstream infections [20]. In this study,half of ST410 were isolated from blood, suggesting that it had enhanced pathogenicity and caused severe infections. This may berelated tothe special virulence factors of LpfA and Gadcodedby genes *lpf*A and*gad*respectively,which were carried only by 14 ST410 isolates different from other STs isolates. The LpfA can mediate attachment to the Peyer’s patches [21–23]. The Gad helps the bacterium to resist the oxidative stress generated by the NADPH oxidase, and the transporteris critically required for proper bacterial phagosomal escape [24]. In addition, there were 21 CREcoisolates which did not produce any reported carbapenemase. These isolates were all ertapenem resistance associated with reduced susceptibility to meropenem and imipenem. Meanwhile, they belonged to 13 STs, and showed distant genetic relationship, suggesting their special genetic characterization. It has been reported that production of ESBLs (e. g. TEM and CTX-M) or AmpC (e. g. CMY-2) in combination with outer membrane permeability defects can be responsible for carbapenem resistance in CREco [15]. Our results showed that combined effect of ESBL/AmpC and OmpF/OmpC proin defects may be the mechanisms that mediated the ertapenem resistance of CREco isolates. Further study is necessary to identify new types of carbapenemases or other related resistance mechanisms inCREco.

## Conclusion

In conclusion, the majority of CREco belonged to ST167, followed by ST410 and ST131, and most of them carried various NDM-coding genes. The spread of high-risk clones of CREco has occurred in different regions of China. Especiallythe high-risk clone groups of ST167 and ST410 carrying*bla*_NDM-5_ have been the most prevalent around China, even could be the main ST types all over the world. Close surveillance is needed to monitor future dissemination.

## Data Availability

All the sequences were uploaded to GenBank with BioProject Accession PRJNA725338.

## Abbreviations

CREco: carbapenem-resistant *Escherichia coli*
SNP: single-nucleotide polymorphism
ST: sequence type
ESBL: Extended-Spectrum β-Lactamase
CRE: carbapenem-resistant *Enterobacteriaceae*
CDC: Center for Disease Control and Prevention
NDMEco: NDM-producing *E.coli*
MICs: minimum inhibitory concentrations
CLSI: Clinical LaboratoryStandards Institute
